# Differential expression of PTEN gene correlates with phenotypic heterogeneity in three cases of patients showing clinical manifestations of PTEN hamartoma tumour syndrome

**DOI:** 10.1186/1897-4287-11-8

**Published:** 2013-07-25

**Authors:** Lorella Paparo, Giovanni Battista Rossi, Paolo Delrio, Daniela Rega, Francesca Duraturo, Raffaella Liccardo, Mario Debellis, Paola Izzo, Marina De Rosa

**Affiliations:** 1 Department of Molecular Medicine and Medical Biotechnology and CEINGE Biotecnologie Avanzate, University of Naples “Federico II”, Naples, Italy; 2 Endoscopy Unit, Istituto Nazionale per lo studio e la cura dei tumori, “Fondazione Giovanni Pascale” IRCCS, Naples, Italy; 3 Colorectal Surgical Oncology - Abdominal Oncology Department, Istituto Nazionale per lo studio e la cura dei tumori, “Fondazione Giovanni Pascale” IRCCS, Naples, Italy

**Keywords:** PTEN hamartoma tumour syndrome (PHTS), Cowden syndrome (CS), Bannayan-riley-ruvalcaba syndrome (BRRS), Sporadic pheochromocytoma, Macrocephaly, *PTEN* tumour suppressor gene, Haploinsufficiency

## Abstract

Cowden syndrome (CS), Bannayan-Riley-Ruvalcaba syndrome (BRRS) and proteus syndrome are disorders known as PTEN hamartoma tumour syndrome (PHTS), that can show remarkable clinical overlap and are all caused by germline PTEN mutations.

We here present two families, one affected by CS and the other affected by BRRS, both carriers of specific pathogenetic missense mutation in exon 5 of PTEN gene, within the catalitic domain. Both PHTS families exhibited extremely variable phenotypes, showing inter- and intra- familial variability. One of the two characterised mutations, the c.320A- > T; p.107Asp- > Val, identified in the CS family, was not previously described in the literature. Furthermore, the BRRS family, carrier of the c.406 T- > C; p.136Cys- > Arg mutation, shows a substantial alteration of PTEN protein expression that well correlates with intra-familial phenotypic variability.

Finally, we describe an apparently sporadic case of an 80-year-old man, with a very low level of *PTEN* mRNA and protein expression, both in healthy and tumour colon mucosa, associated with a very atypical phenotype. He developed a metastatic colorectal carcinoma, macrocephaly and pheochromocytoma.

According to literature data, our observations confirm that PTEN mutations of catalytic domain can cause different syndromes. We suggest that PTEN expression could represent one of the mechanisms involved in the remarkable heterogeneity of the clinical PHTS manifestations within affected families. Furthermore, constitutive strong decrease of PTEN expression in colon normal mucosa could be associated with late onset of colorectal cancer.

## Background

PTEN hamartoma tumour syndrome (PHTS) is the term recently adopted to describe Cowden syndrome (CS), Bannayan-Riley-Ruvalcaba syndrome (BRRS), Proteus syndrome (PS), and Proteus-like syndrome. All these disorders are caused by mutations in the *PTEN* gene and are characterised by extraintestinal manifestations in addition to intestinal polyposis. PHTS is inherited in an autosomal dominant manner, and is likely to be underdiagnosed because of its phenotypic variability, its incomplete penetrance, and also due to the occurrence of many of its component features in the general population [1].

CS is a rare multiple hamartoma syndrome with a reported incidence of 1 in 200,000 individuals. This syndrome is characterised by macrocephaly, mucocutaneous lesions (such as facial trichilemmoma), acral keratosis, glycogenic acanthosis of the esophagus and papillomatous papules. It is also associated with thyroid, breast and endometrial manifestations, including cancer in all of these areas [2]. Renal cancer has also been associated with CS; the risk of developing gastrointestinal carcinoma in CS is still unclear. The BRRS is a congenital disorder characterised by macrocephaly, intestinal hamartomatous polyposis, lipomas, and pigmentated macules of the gland penis; individuals with BRRS should be considered at risk for malignancy, as it is for CS. About 85% of individuals who meet the diagnostic criteria for CS and 65% of individuals with a clinical diagnosis of BRRS have a detectable *PTEN* gene mutation [2,3]. Recently, we reported two affected subjects, with clinical manifestations of PHTS, associated with alteration of *PTEN* mRNA expression, without point mutations within the coding region of the gene [4].

Since the most serious consequences of PHTS relate to the increased risk of breast, thyroid, endometrial and renal cancers, cancer surveillance is of paramount importance in the management of individuals with a PTEN mutation [2,3].

*PTEN* is a 9-exon tumour suppressor gene that encodes for a 403 amino acids protein. It acts as a lipid phosphatase that negatively regulate the PI3K/AKT/mTOR pathway [5,6]. Almost 100 different germ-line mutations of *PTEN* have been reported to date encompassing point, nonsense, frame shift, splice site, missense and deletion/insertion mutations. Most mutations occur in exon 5, but mutations in all other exons, except the first, have also been described [2].

Here we present two families, respectively affected by CS and BRRS syndrome, both carriers of a missense mutation in the catalytic domain of PTEN protein. Because of great phenotypic variability observed in family 2, we performed PTEN protein quantification on peripheral blood cells collected from all subjects of this family. Western blot assay shows that PTEN protein expression well correlates with severity of disease in this family.

Furthermore, we describe a sporadic case showing a very low level of PTEN mRNA and protein expression, both in the healthy and cancer colon mucosa, associated with a very atypical phenotype.

## Methods

### Patients

Samples from all subjects who participated in the study were collected after being granted authorisation from the “Comitato etico per le attività Biomediche – Carlo Romano” of the University of Naples Federico II, with protocol number 120/10. Once the authorisation has been obtained the study has received ethical approval, and participants’ informed and written consent has been obtained.

## Case presentation

### Case 1

The proband 1 was a man affected by Bannayan-Riley-Ruvalcaba syndrome, whose clinical diagnosis was made at the age of 20 years after an endoscopic examination that revealed multiple intestinal hamartomatous gastric polyps and duodenal ulcers.

As previously described [4], polyps found in this family were hamartomas with glandular structures, muscle fibers and also ganglioneuromatosis elements.

At the age of 38 years he developed a colon carcinoma. He also showed macrocephaly with a square cranium and a broad asymmetry of the cerebral hemispheres, glycogenic acanthosis of the esophagus, penis macules, keratosis of the hands and feet and ilo-splenic arteriovenous malformation. The proband's father and brother both showed penis macules; they died of colon cancer at the ages of 80 and 49 years, respectively. As we previously described [4], molecular diagnosis revealed the presence of a missense mutation of *PTEN* exon 5, the c.406 T- > C; p.136Cys > Arg.

His 32 years old daughter was carrier of the same *PTEN* mutation, but she had no clear sign of the disease. She underwent to colonoscopy that revealed the presence of few fundic and duodenal polyps; she was also affected by Hashimoto disease. Secondary amenorrhea occurred at the age of 25 and she showed genitalis macules. Her 31-year-old cousin, also carrier of the pathogenetic mutation, developed only one intestinal polyp.

### Case 2

The proband 2 was a man affected by Cowden syndrome. He showed glycogenic acanthosis of the esophagus, keratosis of the hands and of the feet and only polyps characterised of Juvenile polyps. He later developed a metastatic and locally advanced rectal cancer and hamartomatous small stomach polyps. His daughter, also carrier of the same *PTEN* mutation, developed few hamartomatous colon polyps since the age of 10. She was also affected by Hashimoto disease and arteriovenous malformation and showed small facial keratosis.

### Case 3

In case 3, an 80-year-hold man (proband 3), developed a metastatic colorectal carcinoma. He showed macrocephaly and had previously developed a pheochromocytoma. No other endocrine tumours were referred in their first-degree relatives.

### Molecular analysis of the PTEN gene

Molecular analysis of *PTEN* gene (reported in Methods section, Additional file 1) was performed using a combination of PCR, RT-PCR, sequencing of amplified fragment, dHPLC, computational analysis of mutations with PolyPhen-2 software and SIFT software, Real-Time PCR and Western blot.

## Results and discussion

We performed mutational analysis of the *PTEN* gene, setting-up a combination of RT-PCR reaction of the whole cDNA, PCR of genomic region and sequencing of the amplified fragments.

**Figure 1:**
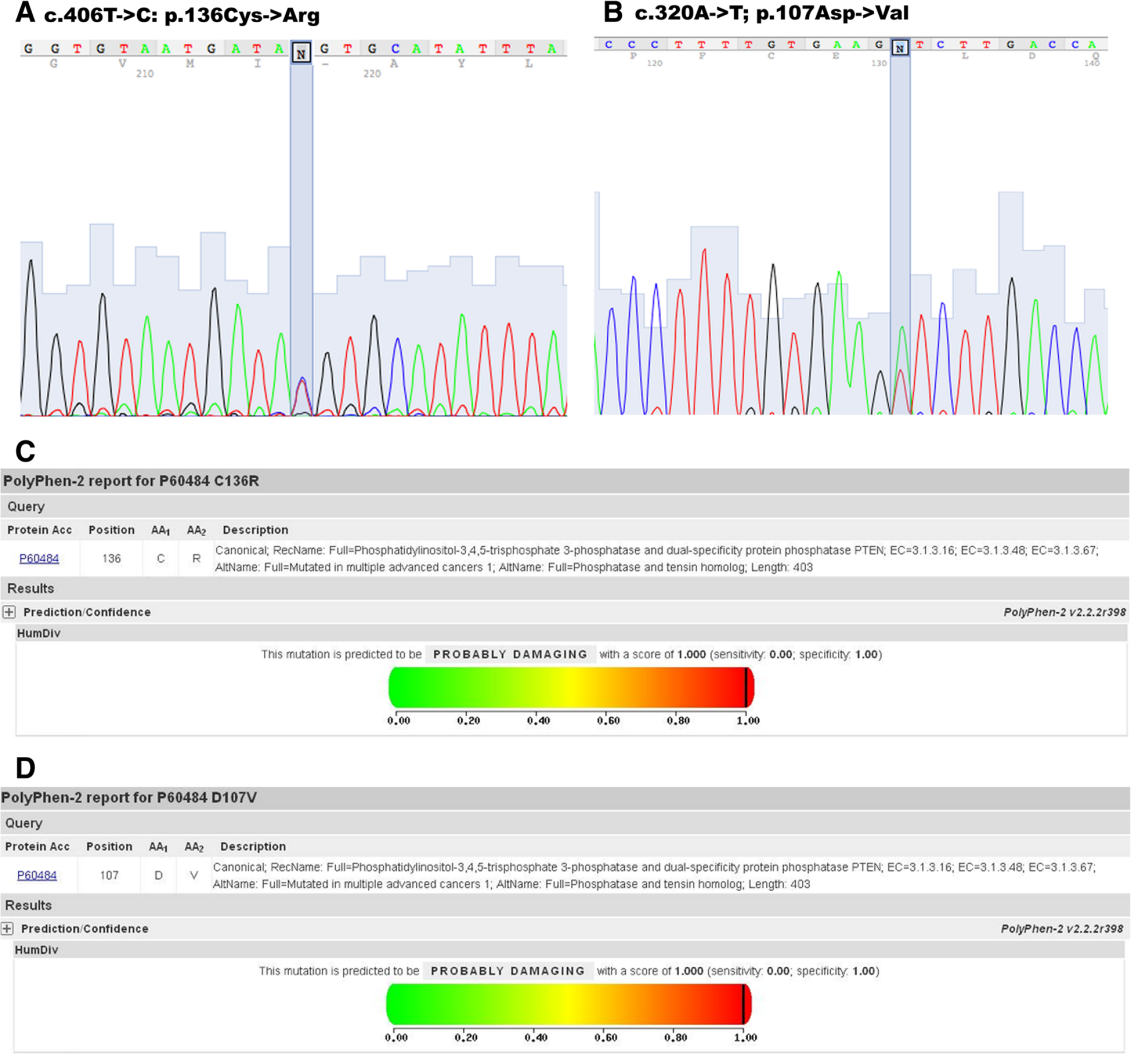
**PTEN exon 5 gene mutations identified in PHTS patients. *A****and****B)****Sequence analysis PTEN exon 5 region.* Sequence analysis was performed on amplified fragments from gDNA of the patients 1 and 2. Reported here are the electropherograms around the identified mutations: c.406 T- > C; p.136Cys- > Arg and c.320A- > T; p.107Asp- > Val. The specific mutated nucleotide is showed within the black box. ***C****and****D)****In sylico analysis of identified mutations.* Graphical representations of PolyPhen-2 computational analysis obtained for mutations c.406 T- > C; p.136Cys- > Arg and c.320A- > T; p.107Asp- > Val.

As we previously reported [4], proband of family 1, affected by BRRS, had a missense mutation named c.406 T- > C, p.136Cys- > Arg in exon 5 of *PTEN* gene (Figure 1A). This mutation, already described in the literature, results in amino acid substitution of cysteine into an arginine residue at position 136 [7,8].

As shown in Figure 1B, proband of family 2, affected by CS, had a missense mutation, the c.320A- > T; p.107Asp- > Val mutation, in exon 5 of *PTEN* gene. This mutation, not previously described in literature, results in amino acid substitution of the asparagine residue into a valine at position 107. These two mutations were predicted to be probably damaging, under computational analysis using PolyPhen-2 software, with a same score of 1.000 (Figures 1C and 1D) and SIFT software with a score of 0. and 0.01, respectively and with a same Median Information Content of 2.52.

**Figure 2:**
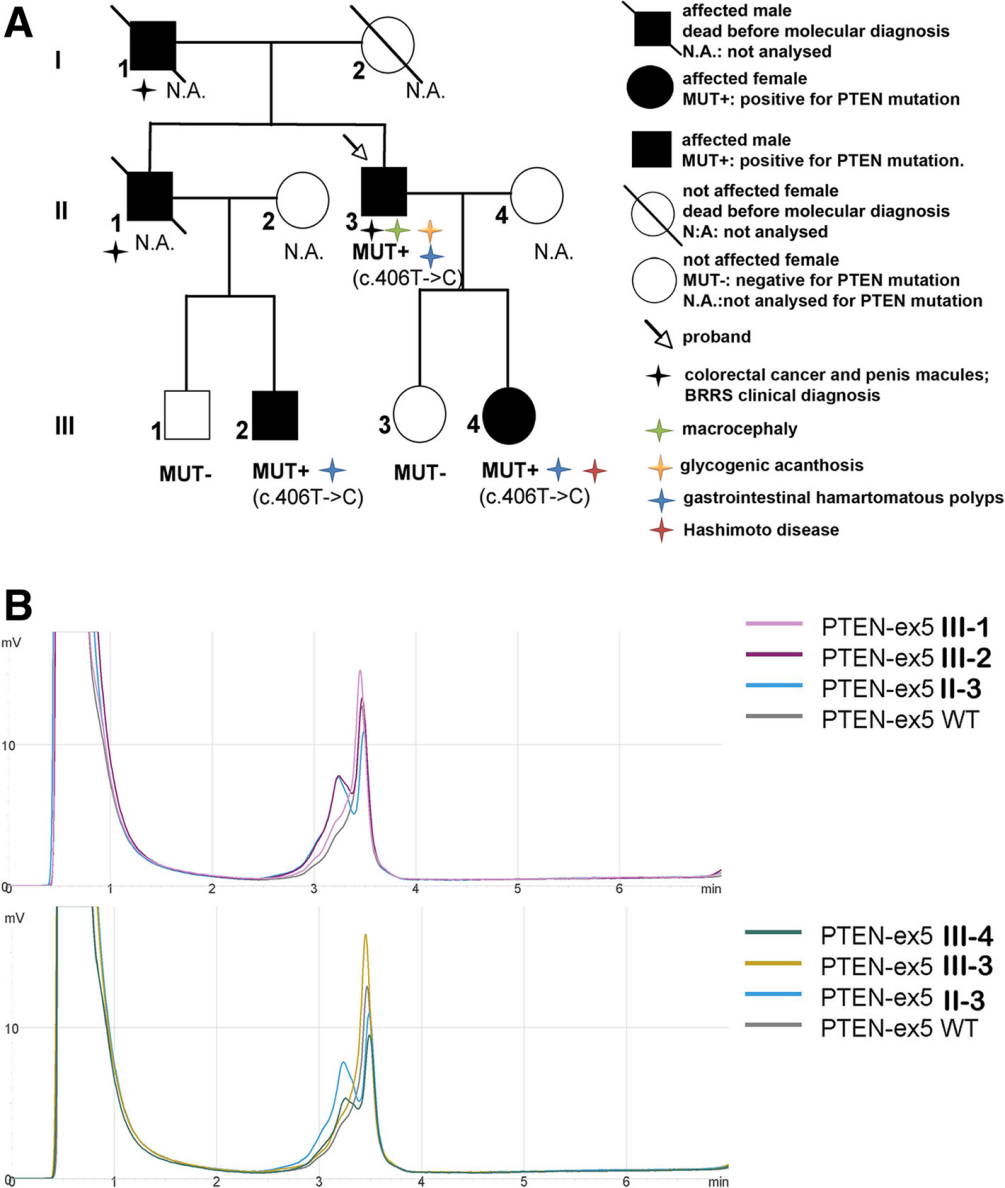
**Molecular analysis of c.406 T- > C; p.136Cys- > Arg mutation within family 1. *A)****Pedigree of family 1.* N.A.: not analysed; MUT+: mutation positive subjects; MUT-: mutation negative subjects. The arrow indicates proband 1 (II-3); III-4: affected daughter; III-3: unaffected daughter; III-2: affected nephew; III-1: unaffected nephew. ***B)****dHPLC analysis within family 1.* dHPLC chromatogram obtained for *PTEN* exon 5 PCR amplified fragment within members of family 1.

**Figure 3:**
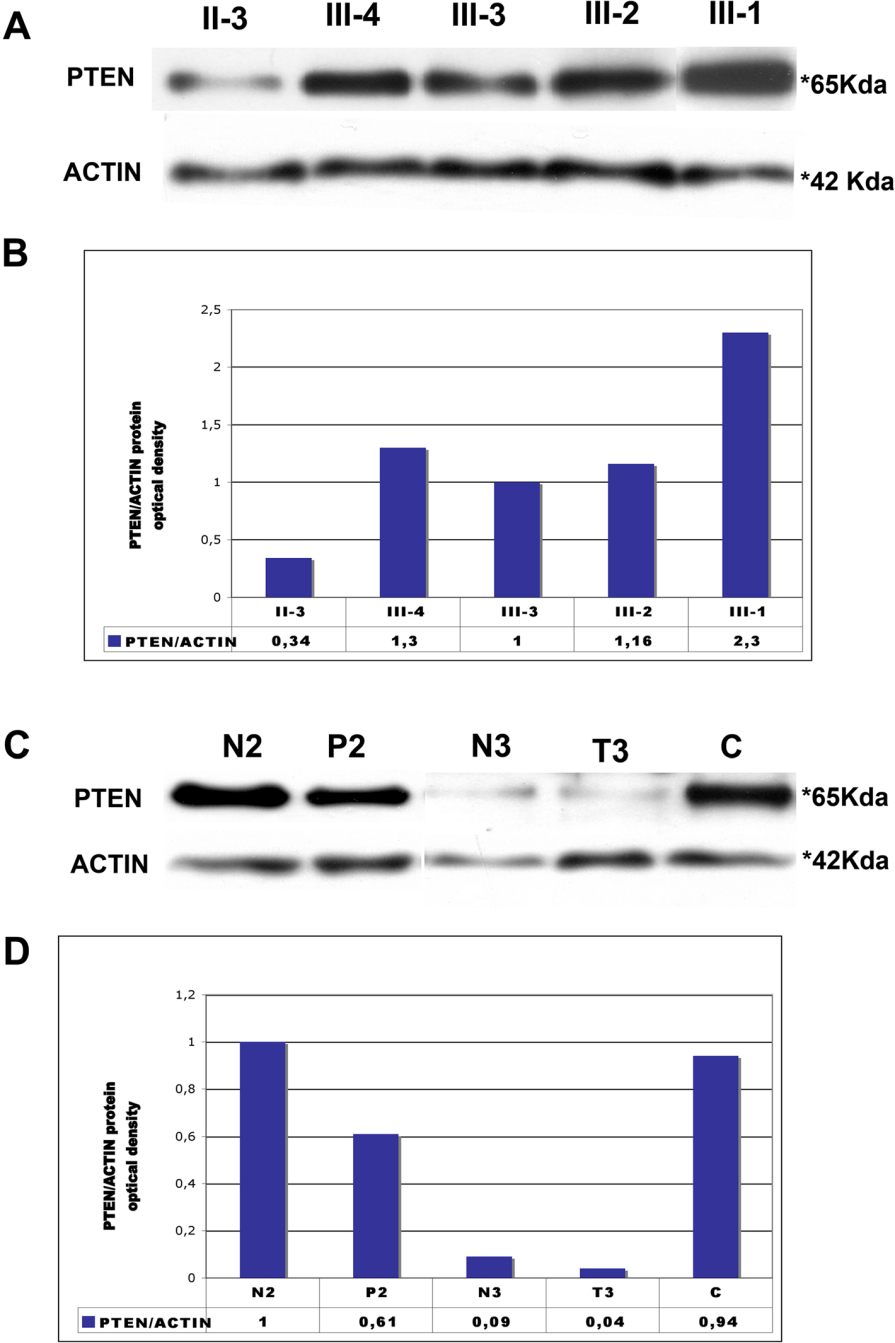
**PTEN protein analysis.** Representative image of three repeated experiments qualitatively similar. **A)***Western blot assay of PTEN performed on protein extracts from peripheral blood cells.* The amounts of PTEN and β-actin were measured by Western blot. II-3, III-4, III-3, III-2, III-1: patients-numbering corresponds to that adopted in the shown pedigree. **B)***Histogram showing optical density of PTEN protein normalized versus actin protein:* Density of the electrophoretic band was obtained with ImageJ software. Relative quantification of PTEN protein normalized versus actin protein was calculated using sample III-3 as calibrator. **C)***Western blot assay of PTEN performed on protein extracts from colon mucosa.* N2: healthy colon mucosa and P2: amartomatous polyp of proband’s daughter described in case 2; N3: healthy colon mucosa and T3: colorectal carcinoma of proband 3; **C**: healthy colon mucosa. **D)***Histogram showing optical density of PTEN protein normalized versus actin protein:* Density of the electrophoretic band was obtained with ImageJ software. Relative quantification of PTEN protein normalized versus actin protein was calculated using sample N-2 as calibrator.

Using dHPLC technique, molecular diagnosis was performed within family 1 and were analysed two daughters and two nephews of the proband. Two of them (subject III-2 and III-4) were carrier of the mutation, as indicated in Figures 2A and 2B. Because of great phenotypic variability observed in family 2, we performed PTEN protein quantification on peripheral blood cells collected from all subjects of this family. Western blot assay shows that PTEN protein expression well correlates with severity of disease in this family. Indeed, the proband, subject II-3 of pedigree in Figure 2A, that exhibited the more severe phenotype among all relatives, also shows the lowest level of PTEN protein expression (Figure 3A). As described in “case presentation” section, he developed a colon carcinoma, showed macrocephaly with a square cranium and a broad asymmetry of the cerebral hemispheres, glycogenic acanthosis of the esophagus, penis macules, keratosis of the hands and feet and ilo-splenic arteriovenous malformation. Instead, his affected daughter and his nephew, respectively subjects III-4 and III-2 of Figure 2A, both carrier of same mutation, did not show unambiguous signs of the disease. Subject III-4, who had a negative colonoscopy, was affected by Hashimoto disease and secondary amenorrhea arising around 25 years. She referred increase of the body weight. Her cousin, subject III-2 of pedigree in Figure 2A, presented only one intestinal polyp. This result suggests that *PTEN* gene dosage could play a role on phenotypic variability observed in this family.

Finally, we have analysed PTEN protein on samples collected from healthy colon mucosa (N2) and hamartomatous polyp (P2) of proband’s daughter described in case 2 and from healthy colon mucosa (N3) and colorectal carcinoma (T3) of proband 3.

As showed in Figure 3B, PTEN protein expression was only slightly lower in the hamartomatous polyp compared to its matched normal mucosa (N2 and P2). Instead, we observed a very low level of PTEN expression both in healthy and colorectal cancer tissue collected from proband 3, compared to healthy colorectal mucosa of unaffected subject (C). Moreover, real time RT-PCR quantification of *PTEN* messenger on normal and colorectal mucosa from proband 3, did not give any signal (data not shown). Overall, these observations suggest that proband 3 could be carrier of a constitutive down-regulation of *PTEN* mRNA and protein. We can not exclude that germ-line alterations in other genes, such as *SDHB, SDHC, SDHD, VHL, or RET*, reported to be altered in patients with apparently sporadic pheochromocytoma [9,10], could be responsible for PTEN somatic inactivation observed in this patient at the level of healthy colorectal mucosa. Unfortunately, he died before we could further analyse genomic DNA extracted from his peripheral blood cells.

It has been demonstrated that PTEN is a bona fide haploinsufficient tumour suppressor gene. A continuum working model, in which subtle variations in the expression of tumour suppressor genes may have a profound impact on tumour susceptibility and progression, has been proposed [11]. In agreement with Alimenti et al. [11], our observations suggest a contribution of *PTEN* gene dosage on phenotypic variability of PHTS patients. In this context, quantitative *PTEN* expression analysis should be extended to point-mutations molecular screening to obtain a more precise interpretation of molecular diagnosis and a better characterization of PHTS patients.

Indeed, PHTS disorders are likely to be underdiagnosed because of their phenotypic variability and their incomplete penetrance [12,13]. However, we can not exclude, as described for other syndromes, that different mechanisms, such as alternative splicing mechanisms [14,15] or allelic variants of modifier genes, could contribute to the observed phenotypic variability [16].

Interestingly, mutants of *PTEN* that maintain partial function may have a selective advantage over mutants that confer a complete loss of function, because complete loss of PTEN induces the activation of a p53-dependent cellular senescence response [17,18]. According with this hypothesis, proband number 3 developed colorectal cancer at the age of 80 years. In our knowledge, this is the first case of CRC patient showing a constitutive strong decrease of PTEN expression in healthy colon mucosa, associated with a colorectal cancer late onset and pheochromocytoma.

## Conclusion

In agreement to recent models of PTEN function described by Salmena et al. [19], we suggest that phenotypes showed in the cases here reported reflect a continuum of functional PTEN loss, suggesting a role for PTEN quantitative regulation on phenotypic variability, either in PHTS families or in apparently sporadic colorectal cancer. Wide population studies will be necessary to better clarify the role of germ-line and/or somatic *PTEN* gene dosage on phenotypic heterogeneity of PHTS patients and apparently sporadic colorectal cancer patients.

## Competing interests

The authors declare that they have no competing interests.

## Authors’ contributions

LP: technical and material support, revision of the manuscript. RM: technical and material support, revision of the manuscript. GBR: technical and material support, revision of the manuscript. PD: technical and material support, revision of the manuscript. DR: technical and material support, revision of the manuscript. FD: technical and material support, revision of the manuscript. RL: technical and material support; acquisition of data; statistical analysis. MD: technical and material support, revision of the manuscript. PI: critical revision of the manuscript for important intellectual content; obtained funding; study supervision. MDR: study concept and design; analysis and interpretation of data; statistical analysis; study supervision; drafting and revision of the manuscript; All authors read and approved the final manuscript.

## Supplementary Material

Additional file 1 **Molecular analysis of the PTEN gene **[20]**.** Click here for file
