# Supplementary material for: Differential expression of PTEN gene correlates with phenotypic heterogeneity in three cases of patients showing clinical manifestations of PTEN hamartoma tumour syndrome
Source: Hered Cancer Clin Pract. 2013 Jul 25;11(1):8. doi: 10.1186/1897-4287-11-8 (PMC3737036; doi:10.1186/1897-4287-11-8)
Supplement: Additional file 1 — Molecular analysis of the PTEN gene [20]. [file 1897-4287-11-8-S1.doc]

***Molecular analysis of the PTEN gene.***

*RT-PCR of PTEN full length coding region in PHTS patients.*

Total RNA was extracted from 3 ml of peripheral blood cells of these three PHTS patients, using Trizol reagent (invitrogen, Life Technologies, Ca, USA), and cDNA was synthesized using 1 μg of total RNA, 500 ng of random hexamers and 1 μl Superscript III reverse transcriptase (Invitrogen, Life Technologies, CA USA), in the presence of 4 μl 5X RT buffer, 1 μl DTT (0.1 M) and 1 mM dNTPs. The reaction was run for 50 mins at 42 °C in a 20 μl reaction volume, heated to 70 °C for 15 mins and quick chilled on ice. Next, 1 μl of the cDNA was amplified by RT-PCR using the following couple of oligonucleotides:

*PTEN*-5’UTR-1FP: TTCCATCCTGCAGAAGAAGC [NM_000314.4],

start +950;

*PTEN*-3’UTR-1RP: TCTGAGCATTCCCTCCATTC [NM_000314.4],

start +2765.

They produce a fragment of 1815 bp of molecular weight. The PCR products were analysed on a 1% agarose gel in a trisacetic acid (TAE)–EDTA standard buffer, and visualized by ethidium bromide staining.

*Sequence analysis of PTEN messenger.*

Sequence analysis of the PTEN full length coding region was performed on RT-PCR fragments of all patients by using the following primer pairs:

*PTEN*-5’UTR2-FP: GCAGCTTCTGCCATCTCTCT [NM_000314.4], start +980;

*PTEN*-7cRP: TCACCACACACAGGTAACGG [NM_000314.4], start +1786;

*PTEN* 5cFP: TTGAAGACCATAACCCACCAC [NM_000314.4], start +1300;

*PTEN*-8cRP: CCTTGTCATTATCTGCACGC [NM_000314.4], start +1971;

*PTEN*-7cFP: CGACGGGAAGACAAGTTCAT [NM_000314.4], start +1728;

*PTEN*-3’UTR2-RP: TAAAACGGGAAAGTGCCATC [NM_000314.4], start +2530.

The analysis was performed in a 3100 Genetic Analyzer (Applied Biosystems, Foster City, CA, USA). For nucleotide numbering, the first A of the initiator ATG codon is nucleotide +1 of the *PTEN* mRNA sequence [GenBank Accession number NM_000314.4]. All oligonucleotides used in this work were designed by using primer-BLAST software

(http://www.ncbi.nlm.nih.gov/tools/primer-blast/).

*Genomic PCR and sequencing.*

Genomic DNA was extracted from 3 ml of peripheral blood cells of PHTS patients and healthy controls, using Nucleon BACC2 Kit (Amersham; Biosciences). Genomic PCR and sequencing of exon 5 was performed for PHTS patients, using oligonucleotides complementary to intronic neighboring boundary regions of the exon

(*PTEN*g5FP: TGTTAAGTTTGTATGCAACATTTCT [NC_ 000010.10], start 89692673;

*PTEN*g5RP: AACCCAAAATCTGTTTTCCA [NC_ 000010.10], start 89693081).

The GenBank Accession number of *PTEN* genomic sequence is: NC_ 000010.10.

*In silico analysis.*

Structure analysis of missense point mutations is very important to understand the functional activity of the mutated protein. For this purpose, we used the servers PolyPhen2 and SIFT [19-20] for investigation of missense variants identified in this study. PolyPhen is available at http://coot.embl.de/PolyPhen/, and predictions are based on a combination of phylogenetic, structural and sequence information characterising a substitution with its position in the protein.

SIFT (Sorting Intolerant From Tolerant) is available at <http://www.blocks.fhcrc.org/sift/SIFT.html> and predicts whether an amino acid substitution affects protein function using sequence homology. It presumes that important amino acids will be conserved in the protein family, and so changes at well-conserved positions tend to be predicted as deleterious. Substitutions with scores less than 0.05 are predicted as deleterious. By default, SIFT builds alignments with a median conservation value of 3.0. Predictions based on sequence alignments with higher median conservation values are less diverse and will have a higher false positive error [20].

*dHPLC analysis.*

A Transgenomic Wave DNA Fragment Analysis System was used to perform dHPLC analysis (Transgenomic Inc., Omaha, Nebraska, USA). Before dHPLC analysis, the PCR products were denatured at 95°C for 5 min and gradually cooled to 20°C using a temperature ramp of 1°C/min on a PCR machine to enable efficient formation of heteroduplex. The mobile phase gradient and running column temperature selected for optimal heteroduplex separation were determined for each amplicon using the Wave Marker 4.4 software provided with the instrument. Aliquots of 8 μl of the PCR products were loaded onto a preheated DNASepVR HT Cartridge column (Transgenomic Inc., Omaha, NB, USA). DNA was eluted at a flow rate of 0.9 mL/min using a linear acetonitrile gradient that consisted of buffer A (0.1 M triethylammonium acetate TEAA-) and buffer B (0.1 M TEAA, 25% acetonitrile). Abnormal elution profiles were identified by visual inspection of the chromatogram on the basis of the appearance of one or more additional earlier eluting peaks.

*Real Time RT-PCR quantification analysis.*

Real-time PCR quantification analysis of *PTEN* messenger was performed on normal colon mucosa (N2) and amartomatous polyp (P2) of proband’s daughter described in case 2 and on normal colon mucosa (N3) and colorectal carcinoma (T3) of proband 3. Healthy colon mucosa from proband 3 were collected far from tumour localization.

Tissues were homogenized and resuspended in Trizol reagent. The *PTEN* mRNA quantification was carried out by amplifying fragments spanning the junctions between exons 5-6, compared to the glucuronidase transcript fragment, using the following oligonucleotides:

*PTEN*-5c2FP: ATGGGGAAGTAAGGACCAGAG [NM_000314.4], start +1495;

*PTEN*-6cRP: TCTTGTGAAACAACAGTGCCA [NM_000314.4], start +1623.

The quantitative Real Time assays were performed using the iCycler iQ Real Time Detection System BIO-RAD. Amplification was carried out within a total volume of 15 μl containing the SYBR Green PCR Master Mix 1X (BIO-RAD), using 20 ng of cDNA. The Real Time PCR reaction was optimized according to the manufacturer's instructions but scaled down to 15 μl per reaction. The PCR conditions were standard (iQ™ SYBR Green Supermix) and all reagents were contained in the standard iQ™ SYBR Green Supermix (BIORAD). The reaction protocol was: 95 °C for 3 mins initial denaturing phase; 95 °C for 15 secs and 60 °C for 1 min. At the end of the PCR, the temperature was increased from 55 to 95 °C at a rate of 3 °C/min, and the fluorescence was measured every 10 secs to construct the melting curve. A nontemplate control was run for each assay, and all determinations were performed in triplicate to ensure reproducibility. Synthesis of the expected PCR product was confirmed by melting curve analysis. Oligonucleotides yielding 100– 150 bp-long PCR products were chosen with an annealing temperature of 60 °C.

*Western blot assay of PTEN protein.*

Total proteins were extracted from 3 ml of peripheral blood cells (about 5-7 x 10^3^/mL cells) collected from all subjects of family 1 and from normal colon mucosa (N2) and amartomatous polyp (P2) of proband’s daughter described in case 2 and from normal colon mucosa (N3) and colorectal carcinoma (T3) of proband 3, using Trizol reagent (invitrogen, Life Technologies, Ca, USA) following the manufacturer’s instructions. Concentrations were determined by using a protein assay kit adopting bovine serum albumin standards, according to the manufacturer's instructions (Bio-Rad Laboratories, Hercules, CA, USA). A total of 50 μg protein was separated by SDS–polyacrylamide gel electrophoresis and blots were prepared on a nitrocellulose membrane Amersham Hybond–ECL (Amersham, GE healthcare, USA). The primary antibody against PTEN was from Cell Signaling technology (Beverly, MA, USA). The antibody against actin was from Santa Cruz (Santa Cruz, CA, USA). The membrane was probed with a secondary antibody against peroxidase-conjugated rabbit and goat immunoglobulin G, and immunoreactive bands were detected using the enhanced chemiluminescence HRP Substrate Immobilon Western, Millipore (Millipore Corporation, Billerica, USA).
